# The Canonical *Notch* Signaling Was Involved in the Regulation of Intestinal Epithelial Cells Apoptosis after Intestinal Ischemia/Reperfusion Injury

**DOI:** 10.3390/ijms15057883

**Published:** 2014-05-06

**Authors:** Guoqing Chen, Zhicao Zhang, Yingdong Cheng, Weidong Xiao, Yuan Qiu, Min Yu, Lihua Sun, Wensheng Wang, Guangsheng Du, Yingchao Gu, Ke Peng, Chao Xu, Hua Yang

**Affiliations:** Department of General Surgery, Xinqiao Hospital, Third Military Medical University, Chongqing 400037, China; E-Mails: maomaoyu1209@163.com (G.C.); zhicao_zhang@163.com (Z.Z.); chengyingdong1964@163.com (Y.C.); weidong.xiao@126.com (W.X.); qiuyuan1988@163.com (Y.Q.); yumimianbao@163.com (M.Y.); slh6260@163.com (L.S.); happywwsh@163.com (W.W.); guangsheng_du@hotmail.com (G.D.); guychao@163.com (Y.G.); pengke620@163.com (K.P.); xuqichao.2007@163.com (C.X.)

**Keywords:** apoptosis, *notch* signaling, intestine, ischemia/reperfusion

## Abstract

*Notch* signaling plays a critical role in the maintenance of intestinal homeostasis. The aim of the present study was to investigate the role of *Notch* signaling in the apoptosis of intestinal epithelial cells after intestinal ischemia reperfusion (I/R) injury. Male C57BL/6 mice were subjected to sham operation or I/R injury. Intestinal tissue samples were collected at 12 h after reperfusion. TUNEL (terminal deoxynucleotidyl transferase-mediated dUTP-biotin nick end labeling) staining showed that intestinal I/R injury induced significantly increased apoptosis of intestinal epithelial cells. Meanwhile, the mRNA expression of *Jagged1*, *DLL1*, *Notch2*, and *Hes*5, and protein expression of NICD2 and *Hes5* were increased significantly after I/R injury in intestinal epithelial cells. In an *in vitro* IEC-6 culture model, flow cytometry analyses showed that inhibition of *Notch* signaling by γ-secretase inhibitor DAPT and the suppression of *Hes5* expression using siRNA both significantly increased the apoptosis of IEC-6 cells under the condition of hypoxia/reoxygenation (H/R). In conclusion, the *Notch2*/*Hes5* signaling pathway was activated and involved in the regulation of intestinal epithelial cells apoptosis in intestinal I/R injury.

## 1. Introduction

Intestinal ischemia/reperfusion (I/R) injury may lead to the development of systemic inflammatory response syndrome, sepsis, and multiple organ dysfunction syndrome (MODS). The loss of intestinal barrier function is considered to be the initial triggering event that turns the gut into the “motor” of MODS [1]. It is known that cell apoptosis is a significant contributor to increased intestinal permeability and there is clear evidence of cell apoptosis in intestinal mucosa after intestinal I/R injury [2–4]. Several signaling pathways, such as PI3K/Akt, ERK1/2, and HIF-1, have been reported to be involved in the apoptosis of intestinal epithelial cells after I/R injury [5–7]. However, the precise molecular mechanisms of this process are still not fully understood.

Studies have shown that the *Notch* signaling pathway plays critical roles in intestinal epithelial cell homeostasis [8]. In mammals, there are four transmembrane *Notch* receptors (*Notch1–4*), two *Jagged*-like ligands (*Jagged1*, *Jagged2*), and three Delta-like ligands (*DLL1*, *DLL3*, *DLL4*) [9,10]. The interaction of these five ligands with *Notch* receptors will activate their proteolytic cleavage at two sites. The cleavage releases the *Notch* intracellular domain (NICD), which translocates to the nucleus and forms an activated transcriptional complex. The complex then activates the transcription of target genes, such as *Hes* (Hairy/Enhancer of split) and Hey (*Hes*-related with YRPW motif), which are two families of basic helix-loop-helix genes [11]. Importantly, the second *Notch* receptor cleavage is mediated by the γ-secretase complex, and inhibition of this proteolytic activity will block the activation of *Notch* receptors [12].

In hepatic I/R injury, interruption of *Notch* signaling resulted in increased intracellular ROS and increased apoptosis of hepatocytes. *Notch2* signal protected hepatocytes from I/R injury by *Hes5* dependent activation of STAT3, leading to the scavenging of ROS [13]. *Notch* signaling protects myocardial cell, endothelial cell, and lymphocyte from apoptosis after injury [14–16]. *Notch* signaling was also reported to protect neuron and non-parenchymal cells from apoptosis in I/R injury [17,18]. After intestinal I/R injury, intestinal epithelial cells undergo proliferation and apoptosis [2–4]. In our previous published study, we detected increased expression of *Notch* signaling components in intestinal epithelium after intestinal I/R injury and showed that *Notch* signaling was involved in the proliferation of intestinal epithelial cells [19]. However, little is known about the role of *Notch* signaling in the apoptosis of intestinal epithelial cells after intestinal I/R injury. The purpose of the present study was to investigate the role of *Notch* signaling pathway in the apoptosis of intestinal epithelial cells after intestinal I/R injury.

## 2. Results

### 2.1. Intestinal I/R Injury Increased Apoptosis of Intestinal Epithelial Cells

**Figure 1.**
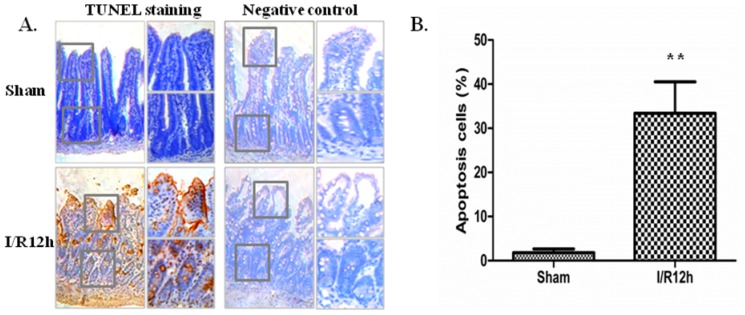
TUNEL-positive cells were increased in intestinal epithelial cells after I/R (12 h). (**A**) TUNEL-positive cells (brown staining with diaminobenzidine) tended to be observed in the intestinal epithelial cells (original magnification ×200). Magnified view of the squared area is shown in the right side of the original picture (original magnification ×400); (**B**) Graphic representation of relative number of TUNEL-positive cells. Data are given as the means ± SDs (*n* = 7). ** *p* < 0.01 *vs.* sham group.

We built the intestinal I/R model through occlusion of superior mesenteric artery (SMA) for 20 min in mice. Jejunum of mice were excised at 12 h after reperfusion. TUNEL examination was carried out to examine the apoptosis of intestinal epithelial cells after intestinal I/R injury. TUNEL staining results showed that the TUNEL-positive cells could be observed in the intestinal epithelial cells of sham operated mice. Importantly, I/R (12 h) significantly increased the number of TUNEL-positive cells compared with sham operation (Figure 1A,B). The TUNEL-positive cells increased 18.6-fold in the I/R (12 h) group compared with the sham group (Figure 1B). However, in the negative control group there was no TUNEL-positive cell (Figure 1A). This shows that all the TUNEL results are credible.

### 2.2. The Notch Signaling Was Activated in a Mouse Model of Intestinal I/R

**Figure 2.**
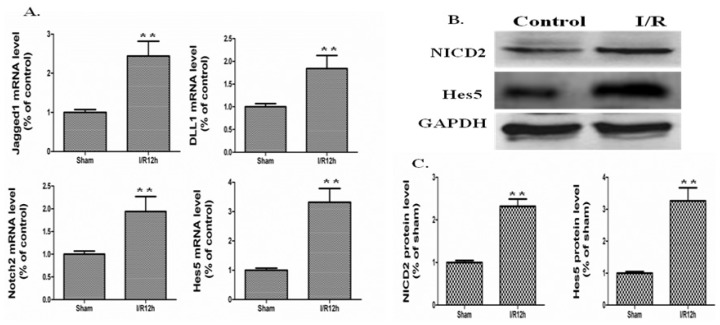
The transcripts and protein levels of *Notch* signaling components were increased in intestine after I/R injury. (**A**) The I/R and sham operated mice were sacrificed at indicated times, mRNA of intestinal mucosa was collected, and real-time PCR was performed to detect mRNA levels of *Jagged1*, *DLL1*, *Notch2*, and *Hes5*. GAPDH was used to verify equivalent loading. Data are given as the means ± SDs (*n* = 7). ** *p* < 0.01 *vs.* sham group; (**B**) Western blot was performed to detect protein expression of NICD2 and *Hes5*. GAPDH was used to verify equivalent loading; (**C**) Graphic representation of relative expression of NICD2 and *Hes5* normalized to GAPDH. Data are given as the means ± SDs (*n* = 7). ** *p* < 0.01 *vs.* sham group.

To investigate the expression of *Notch* signaling components in intestinal epithelium after I/R, we generated a mouse model of intestinal I/R by occlusion of the SMA for 20 min. The clamps were removed, and I/R mice were killed at 12 h after reperfusion. According to published studies, *Notch* signaling components were expressed in intestine of mouse, including four ligands (*Jagged1*, *Jagged2*, *DLL1*, and *DLL4*), four *Notch* receptors (*Notch1*, *2*, *3*, and *4*), and four *Hes* genes (*Hes1*, *Hes5*, *Hes6*, and *Hes7*) [20]. In our experiment, real-time PCR analysis was applied to screen the intestinal epithelium genes for ligands, *Notch* receptors (*Notch1*, *2*, *3*, and *4*) and target genes. As shown in Figure 2A, the mRNA levels of *Jagged1*, *DLL1*, *Notch2*, and the target gene *Hes5* were increased after I/R injury compared to the sham operation group. The mRNA expression of *Jagged1*, *DLL1*, *Notch2* and *Hes5* increased 2.44-, 1.84-, 1.94-, and 3.32-fold, at 12 h after I/R compared to sham operation, respectively (Figure 2A, *p* < 0.01, I/R 12 h *vs.* Sham). At this point, the other *Notch* signaling components, such as *Notch1*, *3*, and *4*, exhibited no significant changes statistically (data not shown). Based on the mRNA expression of *Notch* signaling components, we focused on the protein expression of NICD2 and *Hes5* during the following experiments.

The protein level of *Hes5* also increased after I/R injury (Figure 2B,C). The protein expression of *Hes5* was increased 3.27-fold after I/R injury, respectively (Figure 2C, *p* < 0.01, I/R 12 h *vs.* Sham). As cleavage of *Notch2* is the indicator of *Notch* signal activation, then we investigated NICD2 protein expression. Western blot analyses showed a significant increase in the NICD2 level at 12 h after I/R, indicating the activation of *Notch* signaling (Figure 2B,C). Quantification of the Western blot results revealed a 2.32-fold increase of NICD2 compared with the sham operated mice (Figure 2C, *p* < 0.01, I/R 12 h *vs.* Sham).

### 2.3. Effects of DAPT on the Apoptosis of IEC-6 Cells

In order to investigate the functional role of the *Notch2* signaling pathway in intestinal epithelial cells, we used a culture system with IEC-6 cells receiving 4 h hypoxia, followed by 4 h reoxygenation (H/R), as reported previously [21].

**Figure 3.**
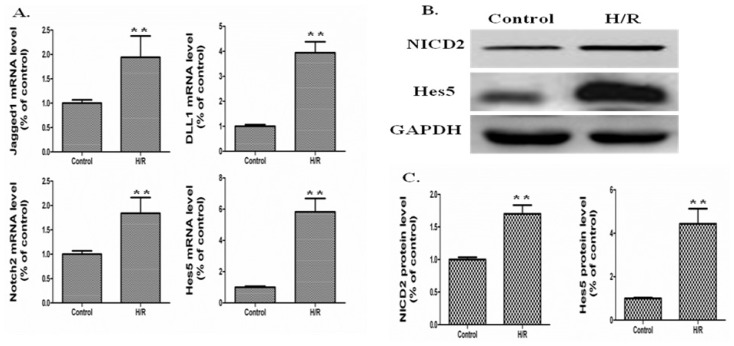
The mRNA and protein expressions of *Notch* signaling components for IEC-6 cells under a hypoxia condition followed by reoxygenation (H/R). (**A**) mRNA expression of *Jagged1*, *DLL1*, *Notch2*, and *Hes5* detected by real-time PCR. The mRNA expression was increased in the H/R group compared to the control group. Data are shown as the means ± SDs (*n* = 5). ** *p* < 0.01 *vs.* control group; (**B**) Protein was extracted from IEC-6 cells. Western blot analyses was performed to detect the protein expressions of NICD2 and *Hes5*. GAPDH was used as the loading control; (**C**) Quantitative analyses of Western blot results were performed for NICD2 and *Hes5*. Data are shown as the means ± SDs (*n* = 5). ** *p* < 0.01 *vs.* control group.

First, we examined if this culture system mimics the *in vivo* I/R model, we investigated the mRNA expression of *Jagged1*, *DLL1*, *Notch2*, and *Hes5* and the protein expression of NICD2, and *Hes5*. The real-time PCR results showed that H/R up-regulated the mRNA expression of *Jagged1*, *DLL1*, *Notch2*, and *Hes5* by 1.94-, 3.95-, 1.84-, and 5.82-fold compared to the control group, respectively (*p* < 0.01, H/R *vs.* Control) (Figure 3A). In accordance with the *in vivo* real-time PCR results, at this point, the other *Notch* signaling components of IEC-6 cells, such as *Notch1*, *3*, and *4*, also exhibited no significant changes statistically (data not shown). Western blot results showed that H/R up-regulated the protein expression of NICD2 and *Hes5* by 1.7- and 4.43-fold compared to the control group, respectively (*p* < 0.01, H/R *vs.* Control) (Figure 3B,C). These results indicated that the IEC-6 culture system mimics the *in vivo* I/R model.

**Figure 4.**
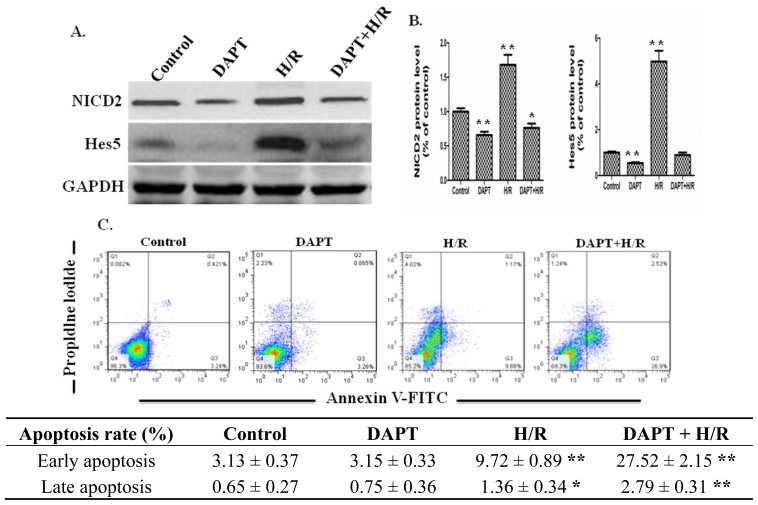
Inhibition of *Notch* signaling by DAPT increased cell apoptosis of IEC-6 cells receiving H/R. (**A**) IEC-6 cells were plated onto a 6-well culture plate and DAPT (20 μM) was added. Protein was extracted from IEC-6 cells and protein expression of NICD2 and *Hes5* was examined by Western blot. GAPDH was used as the loading control; (**B**) Graphic representation of relative expression of NICD2 and *Hes5* normalized to GAPDH. Data are given as the means ± SDs (*n* = 5). ** *p* < 0.01 *vs.* control group; * *p* < 0.05 *vs.* control group; (**C**) Flow cytometry was applied to examine the apoptosis of IEC-6 cells with apoptosis markers (FITC-Annexin V and PI). In the apoptosis map, FITC-Annexin V+/PI+ indicates late apoptosis, FITC-Annexin V+/PI− indicates early apoptosis, and FITC-Annexin V−/PI− indicates normal live cells. H/R and DAPT + H/R increased the early apoptosis and late apoptosis of IEC-6 cells compared to control group. Data are given as the means ± SDs (*n* = 5). ** *p* < 0.01 *vs.* control group; * *p* < 0.05 *vs.* control group.

DAPT is one type of γ-secretase inhibitor that inhibits the activation of *Notch* signaling. DAPT was used to stimulate the IEC-6 cells to investigate the function of *Notch* signaling in intestinal epithelial cells. After DAPT was added to the culture medium of IEC-6 cells for 12 h, IEC-6 cells received H/R or not. After H/R, cell protein was extracted and western blot was applied to examine the the effect of DAPT on the signal transduction of IEC-6 cells. The Western blot results showed that DAPT down-regulated the protein expressions of NICD2 and *Hes5* (Figure 4A,B). Furthermore, we examined the effects of DAPT on the apoptosis of IEC-6 cells receiving H/R or not with flow cytometry. The flow cytometry results showed that H/R increased the early and late apoptosis of IEC-6 cells compared to control group (Figure 4C). Statistically, DAPT had no effect on the apoptosis rate of IEC-6 cells. However, DAPT and H/R together increased the apoptosis rate of IEC-6 cells significantly, especially the early apoptosis of IEC-6 cells (Figure 4C). The results above showed that down-regulation of *Notch2* signaling by γ-secretase inhibition contributed to IEC-6 cells apoptosis under an H/R condition (Figure 4A–C). The *Notch2*-*Hes5* signaling was up-regulated in intestinal epithelial cells under an H/R condition and promoted the survival of IEC-6 cells.

### 2.4 Effects of Silencing RNA for Hes5 on the Apoptosis of IEC-6 Cells

**Figure 5.**
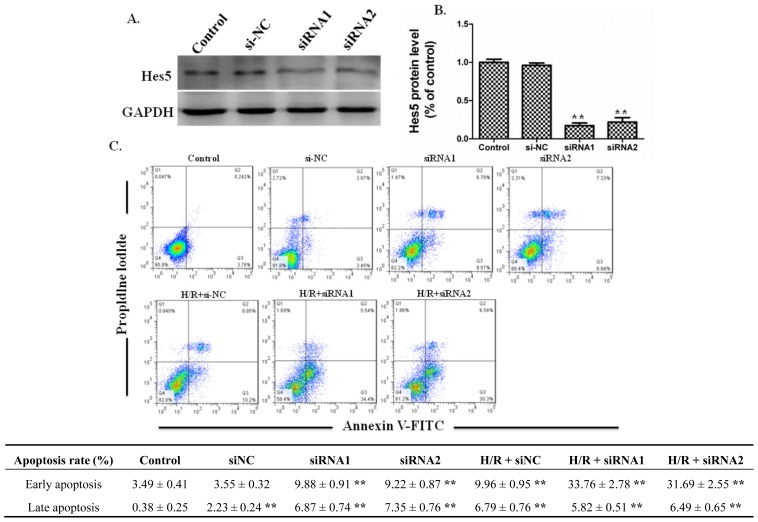
siRNA for *Hes5* increased apoptosis of IEC-6 cells receiving H/R. (**A**) Cells were plated onto 6-well plates. Inhibition of *Hes5* with siRNA was carried out as mentioned in the materials and methods part. After 48 h of culture, protein was extracted from IEC-6 hcells plated on 6-well plates. Western blot analysis showed that siRNA for *Hes5* down-regulated protein expression of *Hes5* compared to the si-NC or control group. GAPDH was used as loading control; (**B**) Graphic representation of relative expression of *Hes5* normalized to GAPDH. Data are given as the means ± SDs (*n* = 5). ** *p* < 0.01 *vs.* control group; (**C**) Flow cytometry was applied to examine the apoptosis of IEC-6 cells with apoptosis markers (FITC-Annexin V and PI). In the apoptosis map, FITC-Annexin V+/PI+ indicates late apoptosis, FITC-Annexin V+/PI− indicate early apoptosis, and FITC-Annexin V−/PI− indicate normal live cells. SiRNA1 and siRNA2 all increased the apoptosis rate of IEC-6 cells receiving H/R significantly. ** *p* < 0.01 *vs.* control group.

To further investigate the effect of *Notch* signaling on intestinal epithelial cell apoptosis, IEC-6 cells were transfected with siRNA for *Hes5*. Western blot results showed that the siRNA for *Hes5* significantly down-regulated the protein expression of *Hes5* (Figure 5A,B). Then flow cytometry was applied to examine the effect of siRNA for *Hes5* on the apoptosis of IEC-6 cells receiving H/R or not. The results showed that suppression of *Hes5* expression increased the apoptosis of IEC-6 cells. Importantly, under the H/R condition siRNA for *Hes5* further increased the apoptosis of IEC-6 cells significantly, especially the early apoptosis of IEC-6 cells (Figure 5C). The results above confirmed that protein expression of *Hes5* could promote the survival of intestinal epithelial cells under the condition of H/R.

## 3. Discussion

In this study, we found that intestinal I/R stress caused increased apoptosis of intestinal epithelial cells. The mRNA and protein expression of *Notch* signaling components were significantly increased in intestinal epithelial cells after I/R injury. Our findings also demonstrated that the *Notch2*/*Hes5* signaling pathway was involved in the protection of intestinal epithelial cells from intestinal I/R injury.

Intestinal I/R injury, which is associated with trauma, hemorrhage, and other shock states, is characterized by epithelial apoptosis, necrosis, and mucosal barrier dysfunction. Studies have shown that intestinal epithelial cell apoptosis is a significant contributor to mucosal barrier dysfunction. Immediate early genes, such as c-*fos* and c*-jun* are involved in the apoptosis of intestinal epithelial cells [22,23]. Some other factors, such as p38 MAPK, IL-6, HGF, and KGF, were also reported to be involved in the apoptosis of intestinal epithelial cells after I/R injury [24–27]. However, the precise molecular mechanisms that accompany I/R within the intestinal epithelial cells need further investigation.

The *Notch* signaling pathway plays critical roles in the maintenance of intestinal homeostasis [8]. Mutations in the *Notch* pathway components, such as the *Notch* DNA binding protein RBP-J or *Notch* receptors will disturb the normal differentiation of intestinal epithelial cells [28–30]. Inhibition of *Notch* signaling activation by γ-secretase inhibitors also lead to disturbed intestinal homeostasis [28–30]. The published studies have shown that the PCNA-positive cells increased at 1 to 6 h after reperfusion and TUNEL-positive cells increased later than PCNA-positive cells in a rat I/R model [22,23,31]. Our previous studies have investigated the role of *Notch* signaling in the proliferation of intestinal epithelial cells [19,32]. In our previous study by our group, we showed that *Notch* signaling was activated and increased the proliferation of intestinal epithelial cells through 6 h after reperfusion [19]. However, in our previous study we did not investigate the *Notch* signaling expression later than 6 h after reperfusion. It is also not clear whether *Notch* signaling was involved in the apoptosis of intestinal epithelial cells after intestinal I/R injury. Thus, in this study we further investigated the function of *Notch* signaling in the apoptosis of the intestinal epithelial cells after I/R injury.

In our study, real-time PCR analysis was firstly applied to screen the intestinal epithelium genes for ligands, *Notch* receptors (*Notch1*, *2*, *3*, and *4*) and target genes after intestinal I/R injury. Physiologically, *Jagged1* and *DLL1* were normally localized in the intestinal epithelial cells [20,33]. In this study, we detected increased expression of *Jagged1* and *DLL1* after I/R injury. To confirm the activation of *Notch* signaling pathway, we further detected increased expression of *Notch2* and *Hes5*. real-time PCR and Western blot results showed that the expressions of *Notch2* and *Hes5* increased significantly 12 h after I/R injury. However, the other *Notch* signaling components, such as *Notch1*, *3*, *4*, exhibited no significant changes statistically. Thus, in the following experiments we focused on the expression of *Jagged1*, *DLL1*, *Notch2*, and *Hes5*. Accompanied with activated *Notch2* signaling, the apoptosis of intestinal epithelial cells also increased significantly. From the above, we aimed to investigate whether *Notch2* signaling was involved in the regulation of intestinal epithelial cells apoptosis after intestinal I/R injury.

To further investigate the functional role of *Notch2* signaling in the regulation of intestinal epithelial cell apoptosis, an IEC-6 cells culture system was applied. In some instances, it may be inappropriate to extend *in vitro* results to *in vivo* conditions; The results showed that the mRNA expression of *Jagged1*, *DLL1*, *Notch2*, and *Hes5* and the protein expression of NICD2 and *Hes5* were up-regulated in IEC-6 cells under the H/R condition. The results above suggested that the *in vitro* IEC-6 cells culture system could mimic the *in vivo* I/R model upon activation of *Notch2*/*Hes5* signaling pathway. Next, the γ-secretase inhibitor DAPT, which inhibits γ-secretase activation of *Notch* receptors, was used to investigate the function of *Notch2* signaling on the apoptosis of IEC-6 cells receiving H/R or not. The results showed that the protein expressions of NICD2 and *Hes5* were down-regulated significantly. The apoptosis of IEC-6 cells was increased significantly when *Notch2* signaling was suppressed by DAPT. Next, siRNA for *Hes5* was used to suppress the expression of *Hes5* and examine the role of *Hes5* in the apoptosis of IEC-6 cells. The results showed that suppression of *Hes5* with siRNA significantly increased the apoptosis of IEC-6 cells. All these results suggested that the NICD2/*Hes5* signaling was involved in the regulation of intestinal epithelial cells apoptosis after intestinal I/R injury.

In summary, our study showed that the *Notch2*/*Hes5* signaling pathway was activated and involved in the regulation of intestinal epithelial cells apoptosis after intestinal I/R injury. Further studies are needed to gain a more precise understanding of the molecular mechanisms of intestinal epithelial cells apoptosis after intestinal I/R injury.

## 4. Experimental Section

### 4.1. Animals

Male, 6–8 week-old, specific pathogen-free, C57BL/6 mice were purchased from the Experiment Animal Center at Daping Hospital of Third Military Medical University, Chongqing, China. All the animal experiments were performed in compliance with the University’s Guidelines for the Care and Use of Laboratory Animals. The protocol was approved by the ethics committee of Xinqiao Hospital, Third Military Medical University, Chongqing, China. Mice were randomly divided into two groups: control group (sham operation, *n* =7) and experimental group (I/R, *n* =7). For the I/R group, the superior mesenteric artery (SMA) was occluded using an atraumatic microvascular clamp for 20 min. Then, we removed the clamps and closed the incisions [19]. Mice were sacrificed at 12 h after reperfusion. The jejunums of mice were quickly removed and processed for histological evaluation, RNA extraction, or protein extraction. The control mice received identical operation procedures without occlusion of the SMA.

### 4.2. Cell Culture

The intestinal epithelial cell line IEC-6, originally purchased from the American type culture collection (Manassas, VA, USA), were grown in Dulbecco’s modified Eagles medium (DMEM, Hyclone, Thermo Fisher, Rockford, IL, USA) supplemented with 10% fetal calf serum (Sigma-Aldrich, St. Louis, MO, USA), 100 μg/mL streptomycin and 100 IU/mL penicillin and cultured overnight for ad*Hes*ion. Once grown, the IEC-6 cells were cultured at 37 °C in either normoxic (20% O_2_ and 5% CO_2_) or hypoxic (1% O_2_ and 5% CO_2_ in a hypoxia chamber) conditions (Thermo Fisher, Rockford, IL, USA). The γ-secretase inhibitor DAPT was added to the medium for 12 h, and the apoptosis of IEC-6 cells were investigated with flow cytometric analysis. Total protein was also obtained for Western blot analysis.

### 4.3. Real-Time PCR Analysis

Total RNA was extracted following a standard isothiocyanate/chloroform extraction method using Trizol (Takara Co., Ltd., Dalian, China), and reverse transcription into first-strand cDNA was performed using the First Strand cDNA Synthesis Kit (FSK-100, TOYOBO CO., Ltd., Osaka, Japan) in the presence of the RNase inhibitor diethylpyrocarbonate (DEPC) (Roche Diagnostics GmbH, Mannheim, Germany) [32]. The amplified cDNA was used as the template DNA for PCR performed with specific primers. In the *in vivo* experiments, the following primers were used: *Jagged1* forward, 5′-CTTGGGTCTGTTGCTTGGTGA-3′ and reverse, 5′-ACATTGTTGGTGGTGTTGTCCTC-3′; *DLL1* forward, 5′-CGGCTTCTATGGCAAGGTCTG-3′ and reverse, 5′-TGTAGCCTCCGTCAGGGTTATCT-3′; *Notch2* forward, 5′-GCGAGCACCCATACCTGACA-3′ and reverse, 5′-TGGGCTGGTGGTCACATCTG-3′; and *Hes5* forward, 5′-AAGCTGGAGAAGGCCGACA-3′ and reverse, 5′-CAGGAGTAGCCCTCGCTGTAGT-3′; GAPDH forward, 5′-AGAAGGTGGTGAAGCAGGCA-3′ and reverse, 5′-AGGTGGAAGAGTGGGAGTTGC-3′. In the *in vitro* experiments the following primers were used: *Jagged1* forward, 5′-CGCCGTGCCCTTTGTGGAG-3′ and reverse, 5′-GGGCCAGACTGCAGGATAAAC-3′; *DLL1* forward, 5′-AGAGGGGCCAACGCTACATGTG-3′ and reverse, 5′-GGCGGAGGCTGGTGTTTCTG-3′; *Notch2* forward, 5′-GGGGGGACCTGCTCTGACTAC-3′ and reverse, 5′-ACGTGCCGCCATTGAAACAGGAG-3′; and *Hes5* forward, 5′-GAAGCCGGTGGTGGAGAAG-3′ and reverse, 5′-CGGCGAAGGCTTTGCTGTG-3′; GAPDH forward, 5′-GGGGCCAAAAGGGTCATCATCTC-3′ and reverse, 5′-AGGGGCCATCCACAGTCTTC-3′; Real-time PCR was performed as previously described [27]. The standard conditions used for real-time PCR were as follows: 94 °C for 10 min, 30 s at 94 °C, 30 s at 60 °C, and 45 s at 72 °C for 45 cycles.

### 4.4. Western Blot Analysis

Tissues and cells were homogenized in cold RIPA buffer (PBS, 1% NP-40, 0.5% sodium deoxycholate, 0.1% SDS, 1 μg/mL PMSF, 1.0 mM sodium orthovanadate, and 1× mammalian protease inhibitor cocktail; Sigma-Aldrich, Shanghai, China). Protein was quantified by the Bradford method using the BCA assay reagent (Beyotime, Shanghai, China). Equal amounts of protein were loaded into SDS-polyacrylamide gels and transferred onto PVDF-Plus membranes. After blocking in 5% fat-free milk for 1 h at room temperature, the membranes were incubated overnight at 4 °C with the following primary antibodies: NICD2 (ab-52302, Abcam, UK), *Hes5* (sc-25395, Santa Cruz, Dallas, CA, USA), and GAPDH (sc-32233, Santa-Cruz, Dallas, CA, USA). The primary antibodies were detected with horseradish peroxidase-conjugated goat anti-rabbit IgG, or goat anti mouse IgG secondary antibody for 1 h at room temperature and detected by the use of a chemiluminescence system (Beyotime, Shanghai, China) and imaging system (Kodak Gel Logic 4000R Imaging System, Carestream, Rochester, NY, USA). A semiquantitative densitometry analysis of the bands were performed using the Kodak Gel Logic 4000R Imaging System (Carestream, Rochester, NY, USA). Protein expression was normalized to the same sample’s expression of GAPDH.

### 4.5. Silencing Hes5 Using siRNA and in Vitro Transfection

To inhibit the *Notch* signaling pathway, an *in vitro* transfection to silence *Hes5* expression was performed. siRNA were chemically synt*Hes*ized by Ribobio (Guangzhou, China). The sequences of the siRNAs targeting *Hes5* were as follows: siRNA1, sense 5′ GCAUUGAGCAGCUGAAACUdTdT 3′ and antisense 3′ dTdTCGUAACUCGUCGACUUUGA 5′; siRNA2, sense 5′ GCAGAUGAAGCUGCUUUACdTdT 3′ and antisense 3′ dTdTCGUCUACUUCGACGAAAUG 5′.

After IEC-6 cells were cultured to 50%–60% confluency in 6-well plates, they were transfected with siRNA at a concentration of 50 nmol for each well using the Lipofectamine 2000 reagent (Invitrogen, Shanghai, China) in antibiotic-free and serum-free Opti-MEM medium, according to the manufacturer’s instructions [19]. An unrelated control siRNA (si-NC) was used in the experiment as the negative control. After 6 h, the medium was replaced with normal IEC-6 cell medium, and the cells were cultured for 48 h. Then, protein was extracted from the cells, and Western blot analysis was performed to detect the protein expression of *Hes5*. Flow cytometric analysis was applied to investigate the effect of siRNA on the apoptosis of IEC-6 cells.

### 4.6. Detection of Epithelial Apoptosis

Tissues were fixed with 4% paraformaldehyde, and 5 μm paraffin-embedded sections were prepared. Intestinal epithelial apoptosis was investigated through terminal deoxynucleotidyl transferase mediated dUTP nick-end labeling (TUNEL) staining using an *in situ* cell death detection POD kit (Roche, Penzberg, Germany) in accordance with the manufacturer’s instructions. As a predealing process, the slides were incubated with 20 μg/mL proteinase K (pH 7.6) for 15 min at room temperature. Then slides were incubated with 0.1% Triton X-100 for 8 min and rinsed with phosphate buffered saline (PBS). Endogenous peroxidase activity was blocked with 3% H_2_O_2_ and methanol. Rinse slides with PBS, add 50 μL TUNEL reaction mixture on sample and incubate for 60 min at 37 °C. Rinse slides three times with PBS and examine the samples under a fluorescence microscope. After this pilot evaluation, the slides were stained by DAB coupling. All slides were counterstained with hematoxylin. As a negative control, the terminal transferase was omitted. The TUNEL-positive cells were counted under a light microscope.

In order to detect the apoptosis of IEC-6 cells, flow cytometric analysis was applied with Annexin V-FITC Apoptosis Detection Kit (KeyGEN Biotech, Nanjing, China) according to the manufacturer’s instructions. IEC-6 cells were cultured as above. Double staining for FITC-Annexin V binding and cellular DNA using propidium iodide (PI) was performed as previously described [34]. The acquisition and analysis were performed using MoFlow (Beckman Coulter, Atlanta, GA, USA).

### 4.7. Statistical Analysis

All the results are presented as the means. The Student’s *t* test was used for the comparisons of the mean values between two groups. ANOVA was used for comparisons of more than two groups. All statistical analyses were carried out using SPSS13.0 software (SPSS, Chicago, IL, USA). *p* < 0.05 was considered significant.

## 5. Conclusions

In this study, we found that intestinal I/R injury caused increased apoptosis of intestinal epithelial cells. The mRNA and protein expressions of *Notch* signaling components were significantly increased in intestinal epithelial cells after I/R injury. Our findings also demonstrated that the *Notch2*/*Hes5* signaling pathway was involved in the protection of intestinal epithelial cells from intestinal I/R injury.
